# A Markerless Pose Estimator Applicable to Limbless Animals

**DOI:** 10.3389/fnbeh.2022.819146

**Published:** 2022-03-28

**Authors:** Vranda Garg, Selina André, Diego Giraldo, Luisa Heyer, Martin C. Göpfert, Roland Dosch, Bart R. H. Geurten

**Affiliations:** ^1^Department of Cellular Neuroscience, Georg-August-University Göttingen, Gottingen, Germany; ^2^Institute for Humangenetics, University Medical Center Göttingen, Georg-August-University Göttingen, Gottingen, Germany

**Keywords:** animal tracker, zebrafish, *Drosophila* larva, gender dimorphism, Hough transform, intermittant locomotion, saccades, undulatory swimming

## Abstract

The analysis of kinematics, locomotion, and spatial tasks relies on the accurate detection of animal positions and pose. Pose and position can be assessed with video analysis programs, the “trackers.” Most available trackers represent animals as single points in space (no pose information available) or use markers to build a skeletal representation of pose. Markers are either physical objects attached to the body (white balls, stickers, or paint) or they are defined *in silico* using recognizable body structures (e.g., joints, limbs, color patterns). Physical markers often cannot be used if the animals are small, lack prominent body structures on which the markers can be placed, or live in environments such as aquatic ones that might detach the marker. Here, we introduce a marker-free pose-estimator (LACE **L**imbless **A**nimal tra**C**k**E**r) that builds the pose of the animal *de novo* from its contour. LACE detects the contour of the animal and derives the body mid-line, building a pseudo-skeleton by defining vertices and edges. By applying LACE to analyse the pose of larval *Drosophila melanogaster* and adult zebrafish, we illustrate that LACE allows to quantify, for example, genetic alterations of peristaltic movements and gender-specific locomotion patterns that are associated with different body shapes. As illustrated by these examples, LACE provides a versatile method for assessing position, pose and movement patterns, even in animals without limbs.

## 1. Introduction

Neuroethology encompasses many behavioral paradigms ranging from complex tasks such as learning and communication (Von Frisch, 1974; Brown, 1976; Dubnau and Tully, 1998; Riley et al., 2005) to more basic activities such as reflexes or locomotion (review: Corthals et al., 2019). Regardless of the complexity of the behavior, behavior is inherently noisy. This noise arises from different internal states of each individual, such as hunger, thirst, or reproductive needs (Abbott, 2020). The noise of the internal states neccessitates repeated measurements and authentic quantification of the examined behavior. Quantifying behavior started with simple observations and written description of animal's behavior (e.g., Yerkes, 1903; Jensen, 1909; Turner and Schwarz, 1914) and developed into artificial intelligence (AI) assisted video analysis (Mathis et al., 2018; Pereira et al., 2018; Werkhoven et al., 2019; Gosztolai et al., 2020).

Most computer assisted methods of video analysis rely on either marker recognition or difference image tracing. Marker recognition filters out physical markers (white balls, stickers, or paint) attached to the animal based on marker properties such as contrast, luminescence, or color (Zakotnik et al., 2004; Spence et al., 2010). Alternatively, marker recognition can exploit the ability of AIs to recognize markers in complex scenes (Mathis et al., 2018; Pereira et al., 2018, 2020; Gosztolai et al., 2020). AIs are able to use visual structures (e.g., limbs, joints, etc.) as markers, obviating the need to attach physical markers. Lightweight animals, however, may neither be able to carry physical markers nor may their bodies bear prominent features that can be recognized by AIs. Markers are also difficult to attach to aquatic or ground-dwelling animals as they might easily be removed by the substrate through which these animals move. In such animals, difference image analysis provides an alternative. Difference image analysis is the basis of LACE, a motion tracker that is presented here. LACE derives the posture from the contour of the animal and is therefore independent of markers. We illustrate the workings and versatility of LACE using two different examples.

In example I, we analyse the peristaltic movement of Drosophila late 3rd instar larvae. The ion channel mutants *nan*^36a^ and *iav*^1^ display disturbed chordotonal neuron function (Kim et al., 2003; Gong et al., 2004; Zhang et al., 2013), causing locomotion and contraction defects (Zanini et al., 2018; Katana et al., 2019). We use these mutants and the wild-type to illustrate the ability of LACE to detect genetic alterations in the body movements of small limbless animals. LACE is also able to differentiate between contraction anomalies and course changes of the animal. This ability relies on the mathematical reconstruction of the antero-posterior axis, which sets LACE apart from other insect motion trackers (Branson et al., 2009; Fontaine et al., 2009; Donelson et al., 2012; Kain et al., 2013; Risse et al., 2013).

In example II, we use LACE to analyse the undulatory swimming movements of zebrafish (*Danio rerio*). Undulatory movement is the principal mode of locomotion in a wide range of limbless animals whose body propagates train of waves that, running laterally from head to tail, propels the animals forward (Gray, 1939). To track such locomotion behaviors, a number of computer-based videography methods have been developed over the past few decades (Fontaine et al., 2008; Green et al., 2012; Maaswinkel et al., 2013; Pittman and Ichikawa, 2013; Pérez-Escudero et al., 2014; Kim et al., 2017; Zhiping and Cheng, 2017; Husson et al., 2018; Walter and Couzin, 2021). These trackers all faithfully report the animal's locomotion behavior, but with variations in focus on larvae (Fontaine et al., 2008; Green et al., 2012), individuals in shoals (Maaswinkel et al., 2013; Pérez-Escudero et al., 2014; Zhiping and Cheng, 2017), single (Geng et al., 2004; Tsibidis and Tavernarakis, 2007; Leifer et al., 2011; Stirman et al., 2011, 2012) and multiple worms (Liewald et al., 2008; Ramot et al., 2008; Swierczek et al., 2011; Wang and Wang, 2013; Brosnan et al., 2021) simultaneous physiological recordings (Kim et al., 2017) or available hardware (Geng et al., 2004; Tsibidis and Tavernarakis, 2007; Ramot et al., 2008; Leifer et al., 2011; Stirman et al., 2011, 2012; Swierczek et al., 2011; Brosnan et al., 2021).

Especially the aforementioned worm trackers (Geng et al., 2004; Tsibidis and Tavernarakis, 2007; Liewald et al., 2008; Ramot et al., 2008; Leifer et al., 2011; Stirman et al., 2011, 2012; Swierczek et al., 2011; Wang and Wang, 2013; Brosnan et al., 2021) are quite similar to our software and surpass its functionality by being able to control lights, camera and in some cases even the x,y-stages of a microscope (compare Table 1). The bodysize of *Caenorhabditis elegans* (ca. 1 mm) makes microscopic recordings necessary. One of the major benefits of behavioral recordings under a microscope is that the background is usually clear and uniformly illuminated. LACE is also capable to detect animals in more complex backgrounds (see Supplementary Material), but has no hardware control integrated.

**Table 1:** Comparison table of LACE to some prominent *Caenorhabiditis elegans* trackers.

**Tracker**	**Representation**	**Code**	**No of organisms**	**Hardware control**	**License**	**Reference**
CoLBert	O,S	Matlab	1	C,T	Free	Leifer et al., 2011
LACE	C,O,S	Matlab	24+	None	Free	-
Multi worm tracker	O,S	C++	<120	C,L	Free	Swierczek et al., 2011
Multimodal illumination	O,S	LabVIEW	1	C,T	Free	Stirman et al., 2011, 2012
Nemo	O,S	Matlab	1	C	Free	Tsibidis and Tavernarakis, 2007
Opto	C	Matlab	<50	C,L	Free	Liewald et al., 2008
Parallel worm tracker	C	Matlab	<50	C	Free	Ramot et al., 2008
Track-a-worm	S	Matlab	1	C,T	Free	Wang and Wang, 2013
Worm tracker	O,S	Matlab	1	C,T	Free	Geng et al., 2004
Wormlab	C,O,S	Closed source	1+	C,L	Comercial	Brosnan et al., 2021

*The representation column shows the type of tracking the software can produce: C notes a single xy point coordinate per animal and frame, called a centroid. S notes a skeleton representation. O notes that the outline of the animal is available. The Hardware Control column shows the ability of the tracker to directly control cameras (C), lighting (L), and microscope x-y-tables (T)*.

BEEtags are lightweight, their handling and application can significantly affect stress levels and behavior in animals. To overcome these obstacles, many other trackers have been developed which are automated and markerless. For instance, Deeplabcut is one such automatic and markerless pose estimator which works on the principle of transfer learning. Though, it provides outstanding results with minimal training data and has been proved successful on multiple species, it does not prove to be equally good for tracing the undulatory movement in limbless animals.

Here, we introduce a tool (LACE) for automated, markerless detection of wave-like movement in limbless animals. The importance of this approach lies in the very fact that it does not consider the organism as a point source or uses any marker to track the pose of the animal, but instead builds a pseudo-skeleton from the contour of the animal. This increases the flexibility of the pose description and circumvents occlusion problems. We illustrate the versatility of LACE by tracking the peristaltic movement of *Drosophila* larvae and undulatory swimming in adult zebrafish.

## 2. Materials and Methods

### 2.1. LACE Limbless Animal TraCkEr

**Figure 1:**
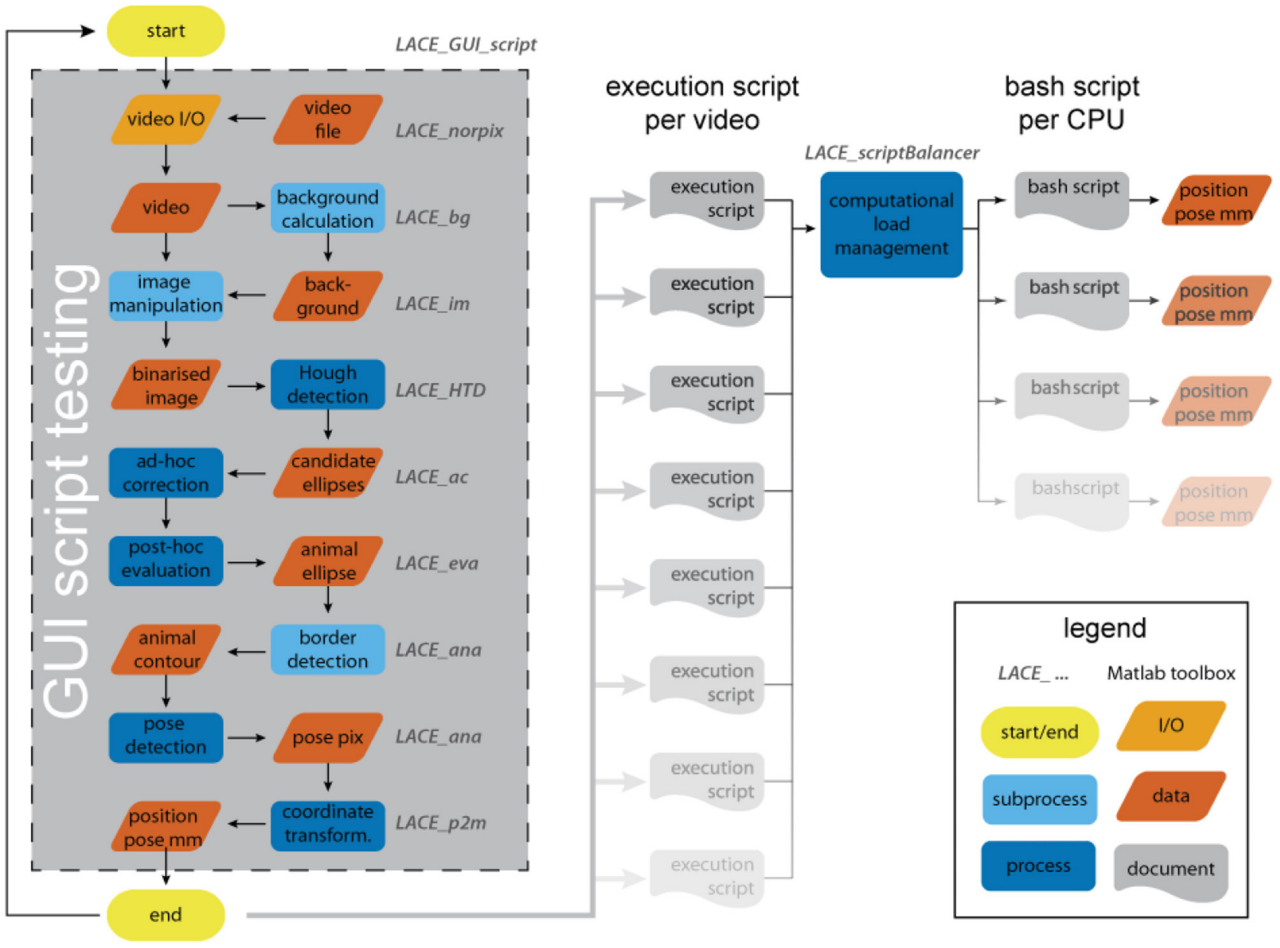
The analysis flow of LACE. The user interacts with most toolboxes through a graphical user interface (GUI). The GUI results in an execution script that holds all information and file positions to run an analysis on the entire video. By testing the script inside the GUI, the system is able to calculate the analysis duration, which is used in the computational load management. The bash scripts can be run over night.

LACE consists of nine toolboxes that solve different tasks: file I/O, background calculation, image manipulation, ellipse detection, *ad-hoc* correction, *post-hoc* evaluation, animal-pose detection, image to world coordinate transformation, and computational load management (see Figure 1). Each of these tasks can be run via the integrated command-line-interface (CLI) of MATLAB or custom graphical-user-interfaces (GUIs).

#### 2.1.1. File Input/Output

LACE can read most video formats through MATLAB's own VideoReader and uses the image manipulation toolbox to load image series, stacks, or single images. We also included a small toolbox (*LACE_norpix* toolbox) that can read in the NorPix Sequence video format (NorPix, Inc., 1751 Richardson Street, Suite 2203, Montreal, Quebec H3K 1G6 Canada), based on the script developed by Brett Shoelson (Mathworks). There is a newer implementation available by Paul Siefert^1^.

#### 2.1.2. Background Calculation

After loading the image sequences or videos, images are prepared to detect the animal. First, one needs to acquire a background image, as a subtrahend for the difference image. The background image can be acquired in different ways: A) If the background is monotone or very stable between recordings (lighting, color, position, etc.), one can record an image without an animal being present. B) In a temporal sequence of images, in which the recorded animal moves through the scene, one can use the differences over time in each pixel to calculate images without the animal being present.

For example, if the animal is dark on a bright background, a maximum intensity projection over time will produce an image without the animal. If the animal is white against a dark background, a minimum intensity projection will provide an empty background. In cases in which the background changes mildly, due to e.g., lighting changes, an average intensity projection might yield the best contrast between animal and background and provide an image of the background without animal. Regardless of the type of projection, these calculations only function as long as the animal does not occupy a subset of pixels all the time, that is when it moves.

LACE offers all three options to calculate your background using the *LACE_bg* toolbox. The *LACE_bg* toolbox includes functions for all image and video formats and is usually called through the *LACE_GUI_script* GUI. As the calculation of the background takes up most computational time, the *LACE_GUI_script* GUI plays a chime at the end of the calculation.

#### 2.1.3. Image Manipulation

**Figure 2:**
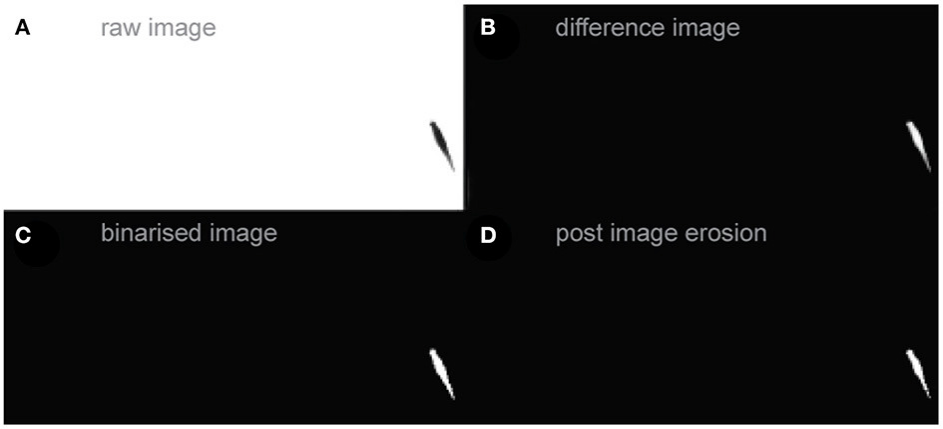
Image Manipulation. **(A)** Raw footage of a zebrafish video. The animal is depicted on the right border of the area. **(B)** Respective difference image. **(C)** Binarised image with a threshold of 0.25 **(D)** Binarised image after erosion and dilatation (image morphology).

After LACE has executed the file I/O and background calculation steps, it performs image manipulation, ellipse detection, and *ad-hoc* corrections frame by frame (see Figure 1). The image manipulation functions are collected in the *LACE_im* toolbox. The purpose of *LACE_im* toolbox is to derive candidate edges of the animal from a given frame and the background. Each frame of the image data is analyzed in 6 steps:

**1. subtracting the background from the frame -> difference image**
  By subtracting the background (see Figure 2A) from the frame (see Figure 2B) all structures of the footage that are not moving (background) are removed while moving objects remain (see Figure 2C).
**2. normalization of the difference image**
  Provided that the animal clearly contrasts with the background, it should be the brightest object in the difference image. The image is normalized to the maximum, assigning pixel values close to 1.0 to the brightest regions of the animal.
**3. binarisation of the difference image -> binarised image**
  The user defines a threshold above which all pixel information is treated as 1 and below which as zero. The resulting image can be seen at Figure 2D.
**4. optional: removal of information outside the region of interest (ROI)**
  The user can define the region in which the animal resides during the video footage. This region is called a ROI (region of interest). All pixels outside the ROI are set to zero (see Figure 2D).
**5. erosion of the binarised image**
  When tracking multiple animals or objects, two moving areas may collide. In such cases, LACE might wrongly recognize two objects as a single one. To avoid this, we use image erosion to remove contact sites of the two animals.
**6. Find edges**
  The edge detection of each animal is done by the Matlab implementation of Canny's edge detector *bwboundaries* (Canny, 1986).

The toolbox also encompasses some simple GUIs for ROI definition. Some standard procedures (e.g., image dilatation, erosion, and rotation) wrap functions of the MATLAB Image Manipulation toolbox (Gonzalez et al., 2004). This allows the user to adjust the procedures without having to interfere with the MATLAB standard toolboxes.

#### 2.1.4. Animal Detection via the Hough Transform

The Hough transformation is a method to test if a given pixel in an image is a part of a certain geometrical shape, such as lines (Duda and Hart, 1972), circles (Yuen et al., 1990), or ellipses (Tsuji and Matsumoto, 1978). The Hough transformation algorithm is fed with a black and white image that only contains bright edges of objects (animals) in a given picture. The Hough transform creates a new image (the accumulator image) in which each pixel of an edge is tested to be a part of one of the aforementioned geometrical shapes. If many points on the given edge belong to the geometrical shape, they will render a bright spot in the accumulator image. The brightness of the spot is relative to the number of pixels that participated in this shape. This allows us to find multiple geometrical shapes inside a given image and rank them by the quality of their detection (brightness of the spot).

Many animals feature a torpedo like body shape, due to aero- or aquadynamic friction. This torpedo like shape can be approximated by an ellipse, which can be detected in the Hough transform (Duda and Hart, 1972; Xie and Ji, 2002). The ellipse detection in LACE (*LACE_HTD* toolbox) wraps the MATLAB implementation by Martin Simonovsky^2^ (Xie and Ji, 2002; Basca et al., 2005). As Hough transform detection is a brute force approach and therefore computational intensive, we use a common simplification: We split the frame into smaller images that only encompass one set of boundaries.

**Figure 3:**
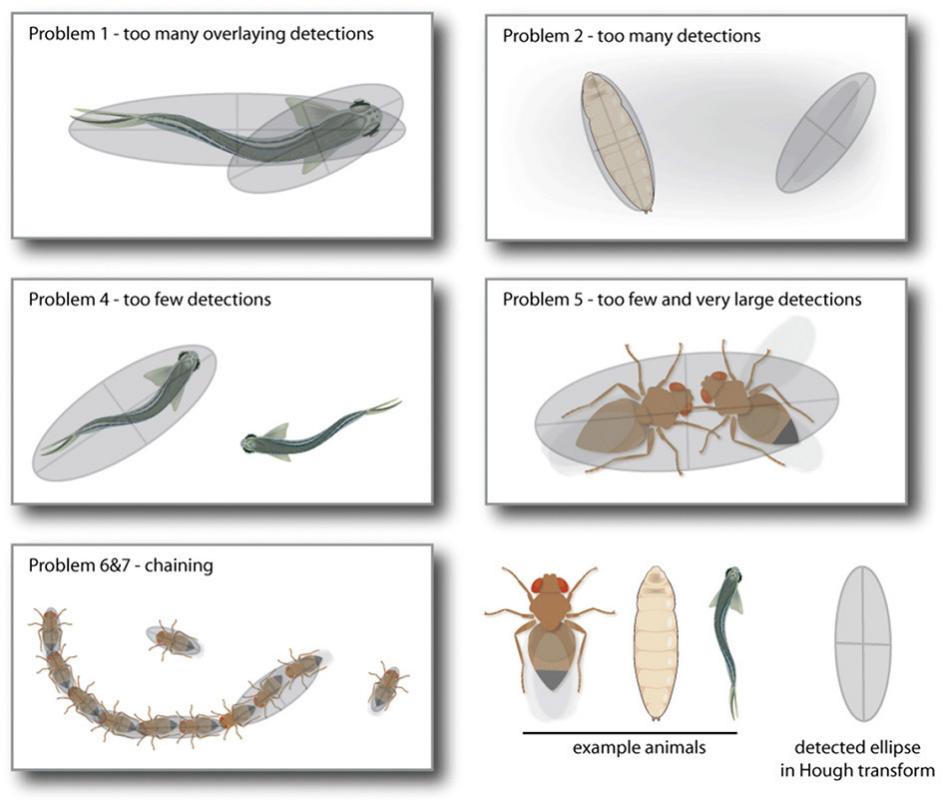
These are illustrations of five standard problems *LACE_ac* toolbox can automatically detect and solve. Problem 1 and 2 are superfluous detections of either the same animal (Problem 1) or other contrast areas in the video frame like shadows (Problem 2). Both are solved by deleting the detection with the lower quality rating. Problem 4 results from one of the detection ellipses not passing all criteria (size, eccentricity, last position) and is solved by taking the detection with the highest quality from the sub-threshold detection list. Problem 5 to 7 are all due to a miss-detection in which two or more animals are lumped together, because of their proximity. These are mainly solved by deleting detections that are too large and choosing from the sub-threshold detection list (Problems 5 and 6) or by splitting up the chain in animal long regions (Problem 7).

Although the Hough transformation is computational intensive, it offers many advantages over classic difference image analysis. Conditions such as maximum and minimum size of the geometrical shape (in our case, the major axis of an ellipse) are already implicit to the detection mode and do not have to be applied *post-hoc*. The orientation of the shape is part of the output of the accumulator space. Even partially occluded geometrical shapes are found, as they still produce a substantially bright spot in the accumulator image. Especially animal interaction often leads to problematic detection situations as the animals occlude each other (see Figure 3) or align so that they become a double wide ellipse (Figure 3). In normal difference image analysis, this needs to be solved manually. The Hough transform results in multiple candidates for these situations, that can be used to solve this problem automatically via *ad-hoc* corrections.

#### 2.1.5. *Ad-hoc* Corrections

Video observations that include multiple individuals can lead to occlusion problems. One major problem is to decide if two animals overlay and therefore create overlaying ellipses or if one animal can be fitted by two overlaying ellipses. Some of these issues can be solved with a prior information that the user provides, e.g., the number of animals present in the observation. This allows LACE to categorize occlusion problems into seven standard problems that the *LACE_ac* toolbox tries to solve.

**1. Problem 1: Too many overlaying instances of detection**
  The Hough transform detection found too many ellipses. The number of ellipses exceeding the user defined number of animals is identical to the number of ellipses with largely overlaying surface area. This indicates a case in which one or more animals are fitted with more than one ellipse. In this case, we keep the ellipse with the best quality of detection from the group of overlaying ellipses.
**2. Problem 2: Too many non-overlaying instances of detection**
  The Hough transform found too many ellipses but none of them overlay. This is rather easy to solve, the ellipse with the lowest detection quality, is deleted.
**3. Problem 3: Problem 1 and 2 occur at the same time**
  First we reduce the overlaying ellipses, if needed, the ellipses featuring the lowest detection quality are deleted afterwards.
**4. Problem 4: Too few ellipses are found**
  In this case, there are no overlaying ellipses but not enough detection was preformed. The Hough transform detection also keeps detection below the quality threshold. We fill up the detection until we reach the number of predicted animals with the best sub threshold quality instances of detection.
**5. Problem 5: Too few ellipses are found but few are larger than a single animal - Chaining**
  We call this problem chaining. If one individual attaches itself to the extremes of the body long axis and aligns itself roughly to the body long axis, this produces a figure eight shape that can be mis-detected as one large animal. From the Hough ellipse detection (see Section 2.1.4), we can estimate if one of the detections is at least 1.5 times larger than a single animal. If this is the case, we split the chain by splitting the oversized detection and refitting ellipses to it with the mean size between minimum and maximum major axis length.
**6. Problem 6: Chaining and not enough instances of detection**
  In this case, solving the chaining problem still can not deliver enough ellipses. In this case, we again fill up the ellipses with the best sub-threshold instances of detection.
**7. Problem 7: The correct number of animals were found, but there is chaining**
  In this case, the chains are refitted as in Problem 5 and the algorithm chooses from all ellipses, the one with the lowest detection value and deletes it until the correct number of ellipses is reached.

Whenever an ellipse-detection is corrected via an *ad-hoc* algorithm, its detection quality is set to –1 to help identify weak instances of detection for later analysis. With the exception of the user provided information, *ad-hoc* corrections employ only information about the current detection frame. Some problems, however, are solved more reliably with information from the detection results before and after the frame in which the problem occurred. These problems are solved by LACE's *post-hoc* evaluation toolbox.

#### 2.1.6. *Post-hoc* Evaluation

After LACE detected ellipses via the *LACE_HTD* toolbox and performed *ad-hoc* corrections (see Figure 1), there might be still some problematic frames left. In nearly all problematic frames, we have a number of candidate ellipses for the animal either above or below the detection threshold. If, for example an animal is not detected in *frame*_*x*_, there are usually a large number of sub-threshold candidate instances of detection to choose from. The *LACE_eva* toolbox uses information from *frame*_*x*−1_ and *frame*_*x*+1_ to choose the best sub-threshold candidate in *frame*_*x*_.

The *LACE_eva* toolbox uses three estimators, which evaluate the detection based on position, surface area, and contour, and then score instances of detection on the basis of their parameter. The user can weigh the scores with factors: For example, if the user wants problematic instances of detection mainly solved via the position of previous instances, he sets the weight of the pose estimator to 1.0 (highest value) and all other estimator weight to relatively low values. Setting the estimator weight to zero omits this estimator for scoring.

**1. Position estimator**
  The position estimator scores possible ellipse detections by the euclidean distance between them and the last detection of the animal.
**2. Surface estimator**
  This estimator scores the candidates by their surface area. Candidates with similar surface area to the detected animal, score higher than those candidates with vastly different surface area.
**3. Contour estimator**
  The contour estimator scores candidates in a similar fashion to the surface estimator, but for the length of the contour.

The evaluation runs automatically and allows so for detection rates of more than 99% during optimal lighting environments (FTIR, Case study I) and over 96% in more difficult lighting environments (Case study II).

#### 2.1.7. Pose Detection

**Figure 4:**
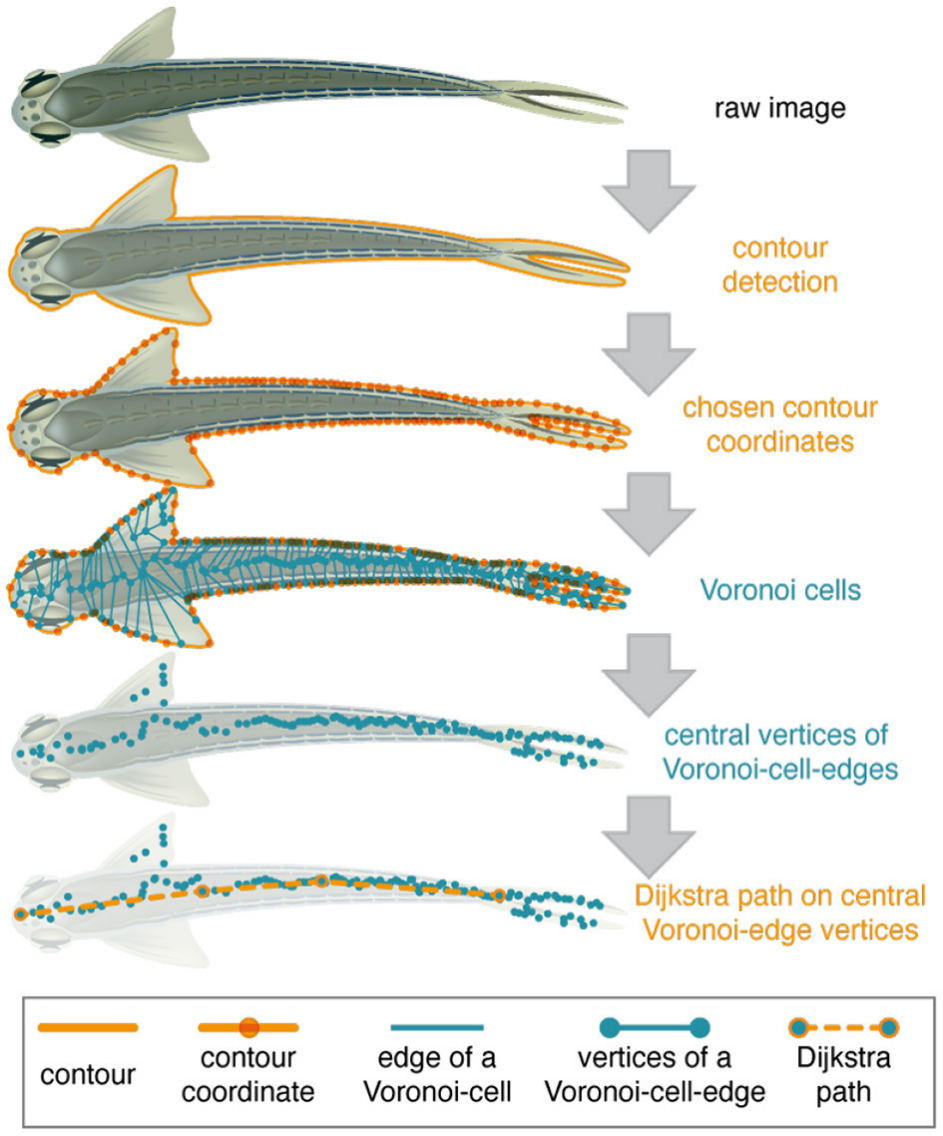
Schematic overview of the pseudo skeleton calculation. The figure illustrates the general procedure used to derive a pseudo-skeleton, therefore all vertices, contours, etc. are schematic drawings and not based on data or results of the algorithms. The pose detection uses the center of the ellipse detected by *LACE_HTD* toolbox as the center for a simple contour detection (solid orange line) via Canny's edge detector. One hundred evenly spaced pixel-coordinates (translucent orange dots) on the contour are chosen. Note that these contour-coordinates are not evenly spaced in the schematic drawing. These contour-coordinates are used as seeding coordinates for Voronoi cells (teal colored lines and dots). A Voronoi cell encompasses all space that is closer to its contour coordinate than to the other contour coordinates. Each cells is enclosed by a number of edges. The Voronoi calculation also generates edges of the Voronoi cells outside the contour of the animal, which are ignored in the algorithm and therefore not drawn here. These Voronoi-edges (teal lines) are represented by their vertices (teal dots). The algorithm selects the vertices that are inside the animal's contour for further computation. Those central Voronoi-edge vertices are now used in Dijkstra's path algorithm to select (teal dots with orange border) the central line along the anteroposterior-axis (dashed orange line).

After LACE detected the animal in the first round via the *LACE_HTD* toolbox and executed automated corrections and evaluations, LACE calculate the pose of the animal *de novo*. The pose detection is performed by the function *LACE_ana_getPose* of the *LACE_ana* toolbox. We return to the edge picture derived from Canny's edge detector (see 2.1.3 step 6). *LACE_ana_getPose* selects 100 evenly spaced pixels from border between the detection object and its background. These pixels are the centers of Voronoi cells (see Figure 1, line 3), which encompass all space that is closer to its center than to the other centers (Dirichlet, 1850; Voronoi, 1908). As a consequence, many new borders and vertices are created inside the silhouette of the object. The vertices are mainly distributed around the mid-line of the object (see Figure 4, line 4).

A Dijkstra shortest path algorithm^3^ is then used on the points inside the detection object (Figure 4, line 4) (Dijkstra, 1959). The start and the end of the path are determined via the closeness to the boundary. *LACE_ana_getPose* then choose shortest path between the start and the end of the mid-line vertices (line Figure 4, line 5). This concludes the detection part of LACE as we detect the animal and know its mid-line.

#### 2.1.8. Coordinate Transformation

Upon this point in the LACE analysis pipeline, all instances of detection and analysis are kept in a pixel coordinate system. In most cases, biologist are more interested in physical measurements. To converge our measurement from pixels to meters, LACE offers two distinctly different types of conversions and a number of measurements. Functions for this transformation can be found in the *LACE_p2m* toolbox.

In all cases, an object of known size is marked inside a frame. These objects can be circular, rectangular or a simple line. In cases of the circles and rectangles, the *LACE_p2m* toolbox interpolates the position of the animal inside the circle or rectangle. Thereby, the coordinate system in which the animal moves, not only changes dimensionality from pixel to meter, but also changes the origin of the coordinate system. For example, if one of the corners of the rectangle is set to (0,0), it becomes the actual coordinate origin. The line measurement only shifts the dimensionality from pixels to meters, but keeps the original coordinate system origin.

#### 2.1.9. Computational Load Management

LACE includes many simple but computational intensive steps such as ellipse detection via Hough transformation (Tsuji and Matsumoto, 1978) or minimal cost matching via the Hungarian algorithm (Kuhn, 1955). Also, it is programmed for CPU usage and therefore has no option to be used on faster and available GPU processors. To avoid blocking a workstation for hours, we have developed a scheduler system.

The general idea is that the user is guided through a graphical user interface (GUI) to define an executable detection script. During the definition of the detection script, the user performs test detection on single images which are later used by LACE to benchmark the computation duration for the whole data set (e.g., a complete movie or image stack). In the second step, the user uses the *LACE_scriptBalancer* to divide the executable scripts on the different CPU cores. As soon as the user does not need the PC anymore, the user can start the detection process and all cores will process the detection scripts. Thereby, you can spend the day recording and defining scripts and run the detection over night.

In a GUI, the user can open the image data (movie, sequence, or image stack) and test the different parameters of image manipulation, such as binarisation threshold, erosion radius, etc. Furthermore, the user has the option to define a ROI and calculate different backgrounds. In the next step, the user can define the parameter of the Hough ellipse detection, such as minimum and maximum length of the major axis of the ellipse or the number of animals depicted in the image data.

The GUI tests the detection parameters and provides the user with an example result on which further refinement can be attempted. In the last step, the user is asked to define a line, rectangle or circle to transform the data from pixel values to meter. Finally, the user needs to define where the detection results should be saved.

Now, the user can save all these parameters as well as the background, file position of the image data, etc., for later use. Also, the GUI writes out a ASCII formatted Matlab script which can be run to analyse the data. As LACE already run several test detections while the user optimized the parameters, it can estimate the computation time per frame. This computation is multiplied by the number of frames in the image data and saved to a MatLab variable called the toDoManager. The toDoManger is a simple cell matrix containing the file position of the executable detection script, the time it has estimated to run (float) and a Boolean variable flagging if the script has already run. The *LACE_scriptBalancer* GUI employs a simple greedy optimisation algorithm (Krumke and Noltemeier, 2009), to balance the computational load of all executable scripts on the available CPU cores. The user can then activate the start script which will activate the executable script for the different cores.

### 2.2. Case I - Larval Locomotion

LACE has been used to study the effect of opsins on the locomotion in *Drosophila* larvae (Zanini et al., 2018; Katana et al., 2019), revealing that these animals require visual opsins for proper locomotion and body contractions. The here published data set illustrates LACE's ability to faithfully track the contractions and locomotion of *Drosophila* larvae.

#### 2.2.1. Locomotion Recordings

An FTIR assay (Risse et al., 2013) was used to assess the locomotory body contractions. Single 3rd instar wandering larvae were recorded crawling on 1% agar with a CCD camera (OptiMos, QImaging, Germany) at 34 frames per second for up to 45s with Micro-Manager. An inverted microscope (IX73, Olympus, Germany) with 1.25X magnification was used for recordings. To keep the larva in frame, the microscope stage was adjusted manually. All larvae were reared at 25°C at 60% humidity in a 12h/12h light-dark cycle on standard fly food (Corthals et al., 2017). CantonS and *w*^1118^ larvae were used to study wild-type control animal peristaltic contractions during locomotion, and *nan*^36a^ and *iav*^1^ mutants that lack the mechanosensory channels NAN and IAV, respectively, and play a proprioceptive role in larval chordotonal neurons, were used to study abnormal locomotion.

#### 2.2.2. Locomotion Analysis

To assess the body contractions, we detected the larva with LACE. Contraction amplitude was calculated as the minimal body long axis (Dijkstra path length from 2.1.7) divided by their maximal length (see 1).


(1) $$\begin{array}{r} A=\left(1-\frac{bodylength_{min}}{bodylength_{max}}\right)\times 100\% \end{array}$$

The curvature index is calculated as follows: The pseudo-skeleton is rotated so that the x-coordinate of both ends equals zero. In a second step, we calculate the integral of the y-coordinates and of the absolute y-coordinates. If both values are large, the animal is performing a turn. If only the absolute value is high, the pseudo-skeleton is in an s-shape form. The integral is subtracted from the absolute integral and therefore the resulting value is always positive. To indicate if it is a left or a right turn, we just multiply the value with the sign of the middle y-coordinate.


(2) $$c=\left(\int_{y=0}^{y=n}\left|y\right|-\int_{y=0}^{y=n}y\right)*-\left(\frac{y_{\frac{n}{2}}}{\left|y_{\frac{n}{2}}\right|}\right)$$

All calculations were performed with MATLAB.

### 2.3. Case II - Zebrafish Locomotion Recordings

We tested the versatility of our tracker by studying the undulatory locomotion in adult zebrafish. This study was performed to evaluate if there are any sex specific locomotion differences between male and female zebrafish. Locomotion videos of 59 adult male and 43 adult female zebrafish were recorded in two different experiments: baseline and startle induced swimming, to make a comparison between their locomotion. For both trials, zebrafish were filmed in a 24.9 x 11.4 cm Plexiglas aquarium with 1.6 cm water depth from above with a high speed camera (Genie HM1024, Dalsa, Imaging Solutions GmbH, Eningen u. Achalm, Germany) linked with a lens system (Optem Zoom 125C 12.5:1 Micro-Inspection Lens System). The setup was illuminated with a LED light plate (Lumitronix) and aquarium light control (Elektronik-Werkstatt SSF, University of Göttingen) from below. For startle induced swimming, a 474 g metal weight was dropped on the setup table, which elicits the recording by closing an open electrical circuit. The fall of the weight was guided by a 13 cm plastic tunnel and produced an impact force of 18.7 N on the surface of the table. The weight collision on the setup table creates a mechanical stimulus which would elicit a certain behavior among individuals. Every trial lasted for 30 s and was filmed with 200 fps. The baseline trials were started 30 s after transferring a fish to the setup tank. Startle induced swimming trials were started immediately after the baseline trials. The recordings were conducted in the diurnal rhythm between 10 a.m. and 8 p.m. For both trials, sequences of the experimental individual without movement for more than 2.5 s were excluded from analysis.

#### 2.3.1. Locomotion Analysis

LACE was used to automatically extract the mid-line position from every single frame. LACE was run on MATLAB R2012b (The MathWorks Inc., Natick, Massachusetts, USA).

## 3. Results

### 3.1. *Ad-hoc* Corrections

**Figure 5:**
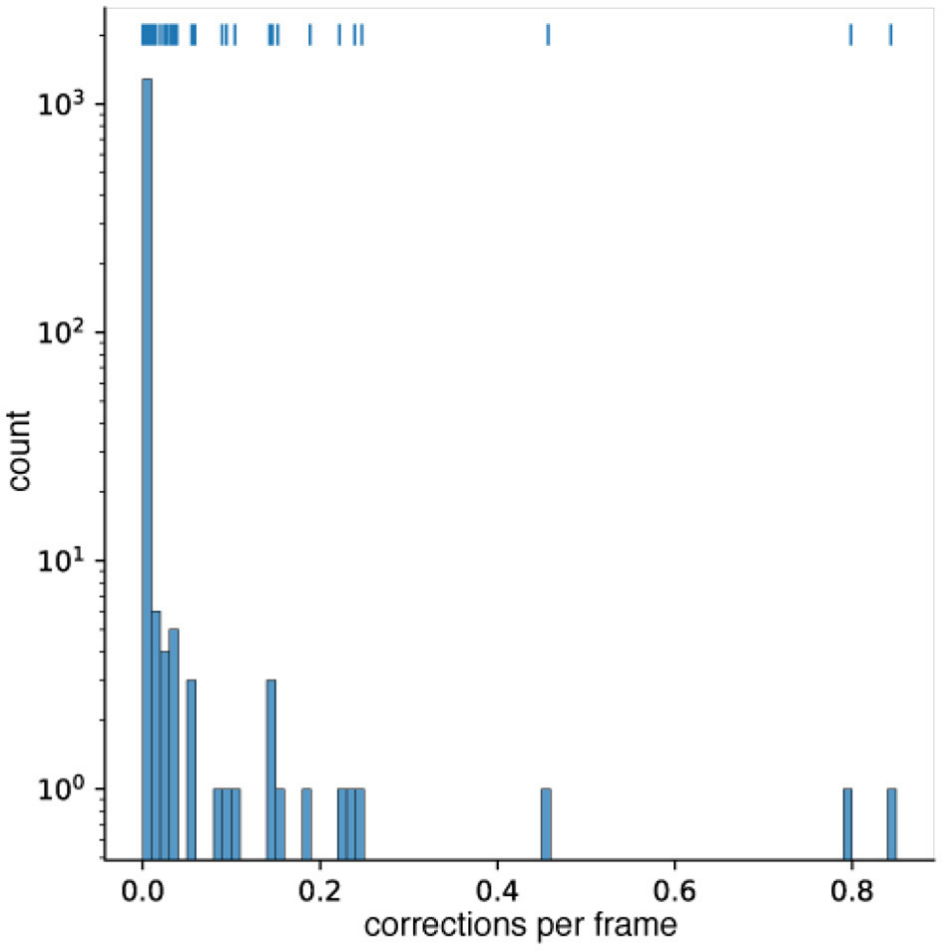
A histogram of the correction frequency per frame for 1,318 different zebrafish video. 1,176 videos needed no correction at all. In 107 videos, less than 5% of the frames were corrected. Note that the counts are depicted on a logarithmic scale. Above the histogram bars, a rug plot (similar to a scatter plot) of the occurrences is given. Each vertical marker represents a video at the given correction frequency on the x-axis.

We analyzed 1,318 movies of zebrafish for the occurrence of *ad-hoc* corrections. In 1,176 (89%) of the videos there was not a single correction needed (see Figure 5). In 107 (8% of all videos) videos, less than 0.5% of their frames needed to be corrected. Whenever the fish made a sharp turn that resulted in a circular form, the algorithm discarded the detection, as it did not fit the expected animal length, this was usually solved by triggering the *ad-hoc* correction from Problem 4. In the remaining 42 movies, up to 80% of the frames needed to be corrected (see Figure 5). The overwhelming reason for this high percentage were wrong user entries. The expected organisms size (in pixel) was set too large or too small so that the detection was dismissed in the first approach. Again the *ad-hoc* correction for Problem 4 was triggered and the correct detection was used.

### 3.2. Case I - Larval Locomotion

**Figure 6:**
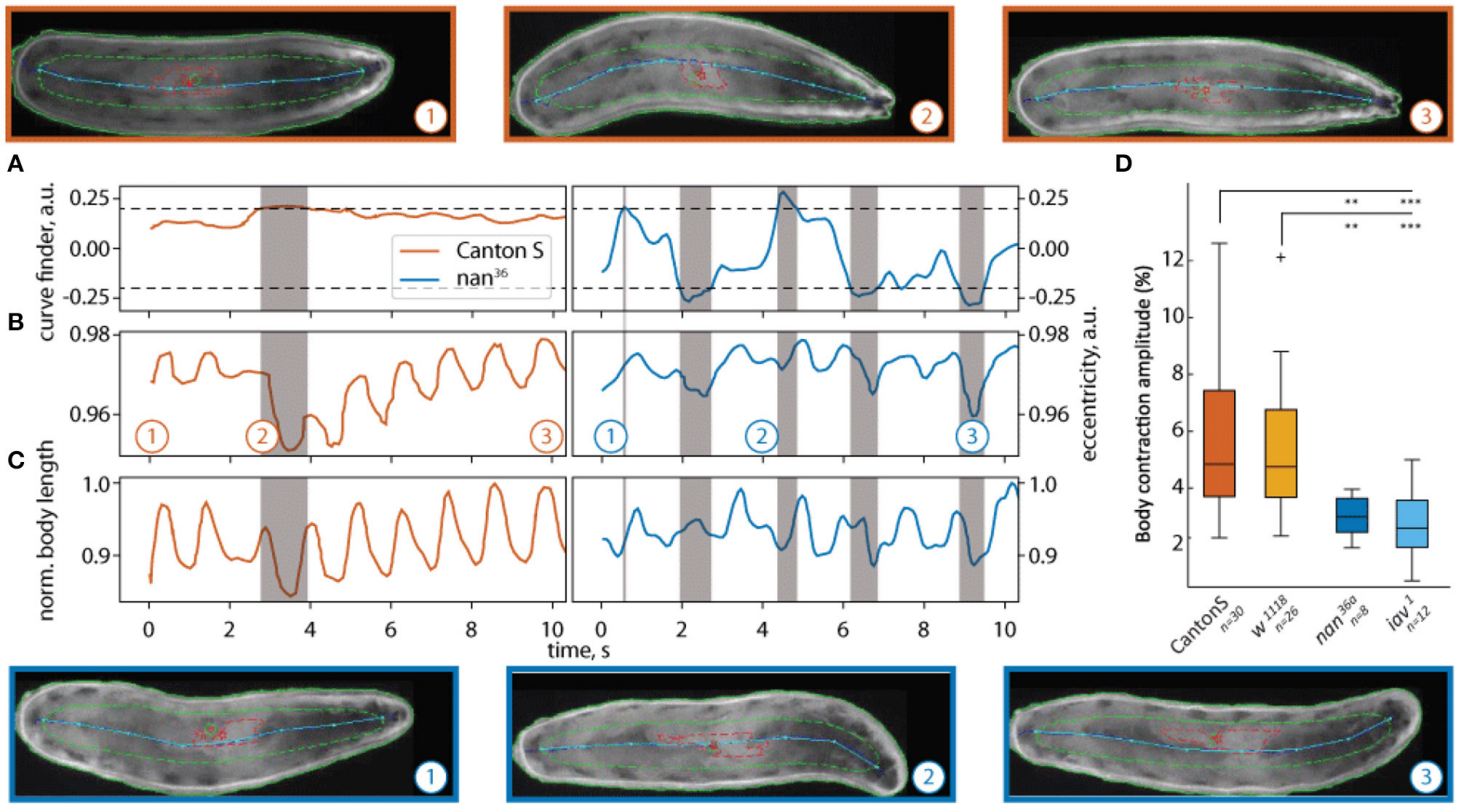
Quantification of body peristaltic contractions of freely crawling *Drosophila* larvae. The results of two trajectories traced with LACE are shown in **(A–C)**: **(A)** the curvature, **(B)** eccentricity, and **(C)** normalized body length of a wild-type (CantonS) larva (orange) and a *nan*^36a^ mutant larva (blue). The curve finder **(A)** detects portions of the video where turning is detected. The turns appear as gray shaded areas (point 2 for CantonS and points 2 and 3 for *nan*^36a^). The white background shows peristaltic contractions during forward crawling (points 1 and 3 for CantonS and 1 for *nan*^36a^). Above (wildtype) and below (*nan*^36a^) still frames from the corresponding times (1,2,3) are depicted. The pseudo-skeleton is superimposed as a light blue line, the contour of the animal is shown as solid green line, the central contour as a dashed green line, and the gut as a red line. Both markers (gut, central contour) were not used in this analysis. In **(D)** the contraction amplitude is quantified for wildtype, *w*^1118^ , *nan*^36a^ and *iav*^1^ mutant larvae. The *nan*^36a^ and *iav*^1^ mutants have significantly lower body contraction amplitudes compared to wildtype CantonS and *w*^1118^. The dataset consists of 30 wildtype larvae (CantonS), 26 *w*^1118^ larvae, 8 *nan*^36a^ larvae, and 12 *iav*^1^ larvae. Statistical significance was tested with Fisher's permutation test on different medians. ***p < 0.001, **p < 0.01.

To assess the efficacy of our tracker, we first studied locomotion in *Drosophila* larvae (Zanini et al., 2018; Katana et al., 2019). When a larva crawls, peristaltic contractions of the body wall muscles lead to shortening and elongation of the body that allows for forward movement (Berrigan and Pepin, 1995; Heckscher et al., 2012). We measured the change in body length during forward locomotion. Phases of turning could easily be detected by the turn detector (2) (see Figure 6A). The body length over time of wild-type larvae forms a regular wave pattern, whereas the body length of the *nan*^36a^ shows an irregular pattern (Figure 6C). The same effect can be seen in the eccentricity of both larvae Figure 6B). Our data revealed that the wildtype and control strains tested have similar body contraction amplitudes (1). Additionally, our analysis showed a significant reduction in the contraction amplitudes in the mechanosensory mutants (see Figure 6D). These effects are in agreement with previous reports of the role of NAN and IAV in Drosophila chordotonal organs (Kim et al., 2003; Gong et al., 2004; Zhang et al., 2013) and the role of these organs in controlling locomotion (Caldwell et al., 2003).

### 3.3. Case II - Zebrafish Locomotion

**Figure 7:**
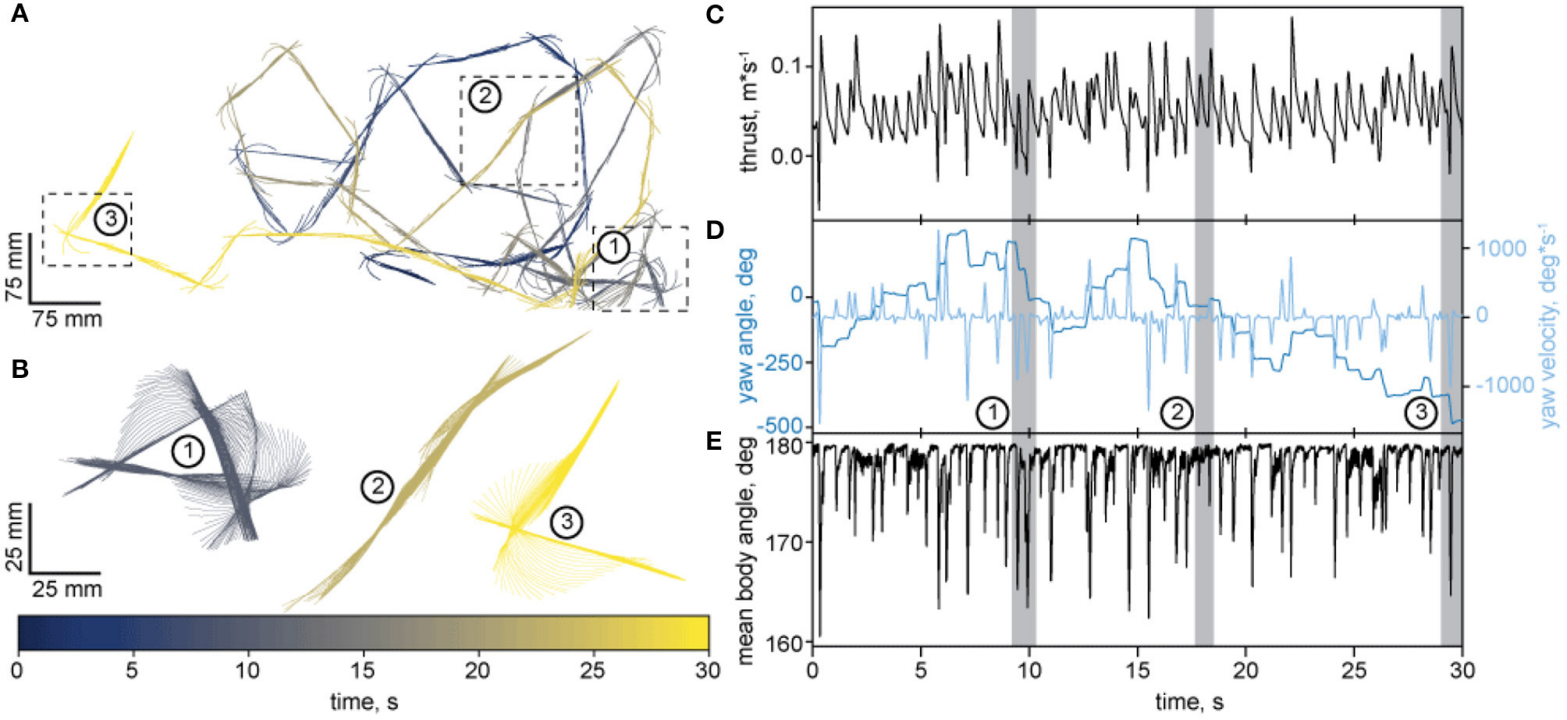
An example trajectory of an adult zebrafish traced with LACE. **(A)** Top view of the trajectory. The body's pseudo-skeleton is plotted as a line every 50 ms. Time is color coded by the color-bar. Three segments of the trajectory were chosen for a close up representation in B. 1 and 3 depicts fast turns and 2 shows a phase of undulatory body wave propulsion. **(B)** Enlarged view of the three segments from A. The pseudo-skeleton is now plotted every 5 ms. Time is encoded by the color bar. **(C–E)** show quantification of the trajectory over time. The gray areas mark the time in which the 3 segments (subplot B) occurred. **(C)** Thrust velocity in m*s^−1^. **(D)** is a YY-plot. The dark blue axis presents the yaw angle in degrees (shown in the same color). The light blue axis shows the yaw velocity in °*s^−1^ (shown in the same color). **(E)** depicts the mean angle of the pseudo-skeleton parts to each other. If the pseudo-skeleton is a perfect line, the angle is 180° and should decrease the more the skeleton is bent.

To further test our tracker, we used adult zebrafish, which propagates undulatory waves along its body during locomotion. Several studies demonstrate sex-specific differences in the activity, anxiety, aggressive and exploratory behavior of zebrafish (Tran and Gerlai, 2013; Ampatzis and Dermon, 2016; Rambo et al., 2017), which all involves locomotion. We thus wondered whether female and male zebrafish might differ in their respective locomotion. To assess this possibility, we analyzed translational and rotational movements during baseline and startle-induced swimming. Figure 7 shows an example of how LACE traces the trajectory of a freely moving fish for 30 seconds. Like many other animals (Kramer and McLaughlin, 2001; Geurten et al., 2017; Helmer et al., 2017), zebrafish move intermittently (compare Figure 7C). Intermittent motion alternates between phases of active propulsion and gliding, which seems to be energy efficient (Kramer and McLaughlin, 2001).

In the example shown in (Figure 7A), the zebrafish separates its movements into rotations and translations (review on the strategy: Corthals et al., 2019). Apparently, zebrafish change their heading when rotating but they also use the rotations for propulsion, as can be seen for the two example turns (segment 1 and 3) in (Figures 7B,C). Each of the orientation turn elicits a spike in thrust velocity (Figure 7C). These spikes are coincidental with pronounced changes in the body yaw (Figure 7D) and bending angle of the pseudo-skeleton/body of the fish (Figure 7E). In addition to this turn-propulsion, zebrafish exploit an s-shaped undulating movement for propulsion shown in (Figure 7B2). The analysis of the pseudo skeleton reveals that although the undulating propulsion elicits similar bending and thrust (Figures 7C,E), there is only negligible change in the orientation of the fish (Figure 7D).

**Figure 8:**
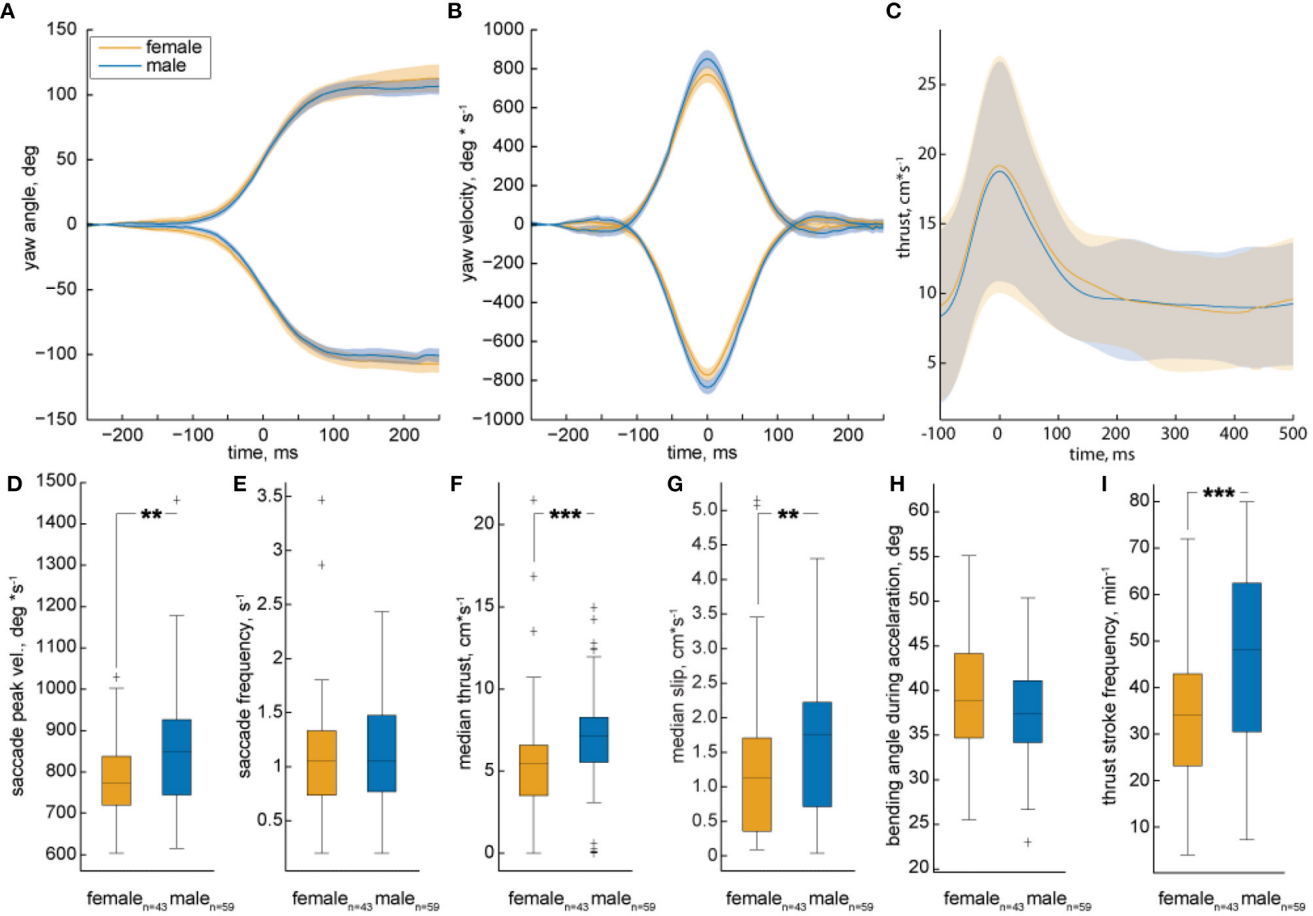
Analysis of multiple trajectories by female and male zebrafish during motivated trials. The median yaw angle **(A)** and velocity **(B)** of turn triggered averages plotted against time. The solid line represents the median of all individuals, shaded areas represents 95% confidence interval. Females are represented by the orange color, males by a blue color. Yaw to the left/right is indicated by positive/ negative numbers, respectively. The yaw angle over time is equal between male and female. Males exhibit higher maximal velocities compared to females. **(C)** The triggered average of all spikes of propulsion is plotted against time. The shaded area represents standard deviation from the mean. There is no significant difference in the propulsion and gliding motion of male and female. **(D–I)** show the quantification of different types of locomotion in the form of box plots. The black line represents the median of all individuals, the box displays the upper and lower quartile, the whiskers denote 1.5 times the interquartile distance and the plus-signs mark the outliers. Color is coded as in A. **(D,E)** The saccadic peak velocity of females as compared to males is significantly lower, while there is no significant difference in the saccade frequency between the two. **(F,G)** The median thrust and slip velocities of male fish are significantly higher as compared to the females. **(H)** There is no difference in the body-bending angle during acceleration. **(I)** There is a significant decrease in the frequency of thrust stroke of females as compared to males. The data set consists of 59 males and 43 females. Statistic significance was tested with Fisher's exact permutation test on different medians. ***p < 0.001, **p < 0.01.

The quantification of many trajectories revealed significant differences between female and male zebrafish locomotion. We analyzed their translational and rotational movements separately and used the peaks in yaw velocity to calculate a triggered average of turning maneuvers (velocity threshold 200°*s^−1^ | see Figures 8A,B). Female fish achieved significantly lower peak turning velocities than males (Figures 8B,D), while they turned as often as males (Figure 8E). The lower peak turn velocities seen in female trajectories might have an influence on the thrust velocity given that turns are also used for thrust-propulsion.

As the fish only accelerates during the propulsion phase of the intermittent motion, the time velocity plot of a trajectory shows distinct peaks (compare Figure 8C). To test for differences in thrust-propulsion, we calculated a triggered average for every peak in the thrust velocity exceeding 10 cm*s^−1^. The mean of these velocity peaks was very similar in male and females (Figure 7C), yet females moved significantly slower than males, as can be seen in the median thrust and slip velocities (Figures 8F,G). This gender dimorphism might reflect differences in body shape and, thus, hydro-dynamic drag. If so, we expected to see differences in the gliding phase after a thrust stroke (Figure 8C), but gliding velocities were the same for females and males. Differences in body shape might cause difference in body bending, yet also bending seemed to be the same (Figure 8H). The significantly different thrust velocity is caused by a significantly different thrust stroke frequency (Figure 8I). As the turn frequency is similar between the sexes, we can deduct that the significant thrust-stroke-frequency difference is caused by a higher frequency of s-shape propulsion.

## 4. Discussion

The detection of animals in videos is the basis for many neuroethological studies, ranging from locomotion analysis (Muybridge, 1882) to learning tests (Barth et al., 2014). Methods that facilitate the tracking of animals are evolving constantly, facilitating the analysis of large sets of behavioral data. Currently, most trackers fall into two categories: trackers that treat the animal as a solid object with an orientation (Branson et al., 2009; Donelson et al., 2012; Pérez-Escudero et al., 2014; Geissmann et al., 2017; Mönck et al., 2018; Rodriguez et al., 2018; Werkhoven et al., 2019; Krynitsky et al., 2020) and trackers that represent the animal as a skeleton (Fontaine et al., 2009; Kain et al., 2013; Nath et al., 2013; Mathis et al., 2018; Pereira et al., 2018; Gosztolai et al., 2020). The application of such trackers to limbless or rather featureless animals is sub-optimal because skeleton representations are based on readily identifiable body parts that can be used as visual markers (e.g., joints,legs,antennae) or require the attachment of physical markers, which is not always possible. The representation of animals as solid lines or single points also discards important features of the trajectory, as limbless animals generate propulsion by deformation of their bodies.

The tracker LACE has been specially designed for tracking limbless animals, though it can be applied to other organisms as well. Since limbless animals usually lack clear markers such as color patterns or joints, arms, and legs, pose estimation requires information about the mid line of the body. LACE estimates this mid-line from the contour of the animal and treats this mid-line as a pseudo-skeleton that allows to quantify body deformations without using physical or visual markers. To our knowledge, the only available tracker that represents animals in a similar fashion is FIM-track (https://github.com/i-git/FIMTrack) which allows to analyze animal trajectories using frustrated total internal reflection (FTIR) (Risse et al., 2013). FIM-track was developed specifically for analyzing FTIR trajectories and we found it to be less efficient under different lighting conditions. For example our fish tanks were back lit and therefore the signal to noise ratio, was significantly lower than in an FTIR experiment.

LACE consists of nine toolboxes that can be used as a stand-alone software or can be combined with other existing trackers. The pseudo-skeleton generator, for example, can be used in combination with other trackers that can detect the contour of the animal (Fontaine et al., 2009; Nath et al., 2013; Risse et al., 2013). The video loading module of LACE can read nearly any standard file format and works for different lighting conditions. This *LACE_bg* toolbox offers the advantage of calculating background images for varying light-dark conditions. LACE also allows one to define the region of interest (ROI), allowing to discard irrelevant information. Although already available software (e.g., Fiji Schindelin et al., 2012, 2015) could have been used to create ROIs, we wanted to integrate everything into one GUI for ease of use. The *ad-hoc* and *post-hoc* evaluation toolboxes allow to record multiple animals or objects together, automatically solving many occlusion problems. LACE provides the *LACE_p2m* toolbox to convert pixel coordinates into arena-based SI coordinates. Normally, these computational intensive steps takes hours. To speed up analysis, LACE is equipped with a scheduler system that allows for a division of labor between different CPU cores, allowing users to record data and define scripts during the day and run the analysis overnight. Many of those features can be found in other tracking software, but not in the same combination.

The most comparable trackers to LACE are trackers of the model worm *Caenorhabditis elegans* (see comparison Table 1) (Geng et al., 2004; Tsibidis and Tavernarakis, 2007; Ramot et al., 2008; Leifer et al., 2011; Stirman et al., 2011, 2012; Swierczek et al., 2011; Brosnan et al., 2021). This is not surprising as *Caenorhabditis* is a limbless organism with very few distinguishable anatomical markers. Although recording videos from a microscope has disadvantages (e.g., moving the stage, low photon yield, etc.) which many of the mentioned trackers overcome elegantly, there are certain advantages. Two of those advantages are uniform background and iso-illumination across the field of view. LACE handles more complex lighting situations as well (see Supplementary Material).

The most comparable fish tracker is idTracker (Pérez-Escudero et al., 2014; Romero-Ferrero et al., 2019). idTracker shares most of LACEs features and has a much more sophisticated detection of individual organisms in a group. LACE identifies individuals via their position, direction, and posture. In contrast to LACE, idTracker identifies the individuals by the eigenvalues of their Gestalt. To our knowledge idTracker does not however derive a pseudo-skeleton for the identified individuals, which is crucial to most of our analysis.

LACE is rather computational intensive and therefore cannot track multiple animals in real-time. Many of the aforementioned worm trackers and the TRex tracker (Walter and Couzin, 2021) have real time capabilities. Whereas the worm trackers can rely on slim algorithms due to bright and uniform backgrounds, TRex achieves this computational speed by using a non interpreted language (C++). LACE is written in a less efficient but more accessible programming language (Matlab), which allows the user to customize the source code directly.

We have illustrated the versatility of LACE using crawling Drosophila larvae and swimming adult zebrafish as examples. Studying peristaltic contractions during locomotion of *Drosophila* larvae allows to screen for genes, neurons, and networks involved in proprioception, mechanosensation, and locomotion (Caldwell et al., 2003; Hughes and Thomas, 2007; Zanini et al., 2018; Katana et al., 2019). In Zanini et al. (2018) we used LACE to analyse the role of opsins in mechano-transduction. We used LACE instead of the FIM-Track because we needed to analyse locomotion under infrared-light and visible light conditions. Although FIM-Track worked well with infrared light, it did less so in the presence of visible light. A costly infra-red pass filter would have solved this problem. Another option seemed to express green-fluorescence protein (GFP) in the larval muscles, as was done to study the roles of mechanosensory neurons during peristalsis (Hughes and Thomas, 2007). Our tracker bypasses the need for GFP expression and can be used to track many animal species. Its ability to detect turning events during locomotion allows to analyse exclusively, for example, periods of forward locomotion. LACE can precisely track this locomotion and distinguish turns from normal peristaltic movements. It also allowed us to identify subtle changes in locomotory body movements in mechanosensory mutants (Zanini et al., 2018).

By using LACE to track zebrafish, we tested for differences in locomotion between adult females and males. Several studies had indicated sex specific differences in different forms of zebrafish behavior (Philpott et al., 2012; Tran and Gerlai, 2013; Ampatzis and Dermon, 2016; Rambo et al., 2017), yet whether these differences extend to locomotion, had, to the best of our knowledge, not been explored. Using LACE, we found that females swim slower than males and turn less fast (Figures 8D,F,G). Possibly, the ovary makes it more difficult for the females to bend their body during turning. We did not find any sex-specific differences in the bendability (Figure 8H), yet visual inspection of fish revealed that females with full ovaries are larger than males. The more slender body of males presumably experience less drag in water, but thrust strokes and declines were virtually identical for the two sexes, arguing against pure effects of drag (Figure 8C). The same argument holds true for a difference in inertia caused by a difference in weight between both sexes [males 0.23 g, females 0.36g (Eaton and Farley, 1974)].

Even though a female has to overcome a higher inertia to change its velocity (2^nd^ law of motion Newton, 1833) the resulting velocity profile is nearly identical (Figure 8C). When we analyzed the frequency of thrust strokes, we found that males perform more thrust strokes in a given time period, allowing them to swim faster than the females. Moreover, while the turning frequency is nearly identical for the two sexes, males more often perform s-shape thrust strokes, propelling them forward with higher speed. LACE has the potential to reveal such minute but crucial information from the video data without a need for any markers or AI-training. Overall, by this study, we have shown that LACE has the capability to differentiate between different aspects of locomotion ranging from fast turns to bendability and forward motion, revealing a hitherto undescribed behavioral sexual dimorphism in zebrafish.

LACE is a simple, markerless, and fully automated tracker for studying undulatory locomotion in limbless animals. We have demonstrated that this tracker can be used to study different aspects of locomotion behavior in different types of limbless organisms and in more complex lighting environments. Our results indicate that LACE has the potential to reveal novel aspects of locomotion behavior in a variety of larger organisms. We hope that our tracker will facilitate the study of movements and pose in various animals species.

## Data Availability Statement

The original contributions presented in the study are included in the article/Supplementary Material, further inquiries can be directed to the corresponding author. LACE source code is available at https://github.com/zerotonin/LACE.

## Ethics Statement

Ethical review and approval was not required for the animal study because this was just video recordings of unaltered fish.

## Author Contributions

VG, SA, and LH performed experiments on zebrafish. DG performed the experiments on *Drosophila*. BG wrote the code with inputs from all other authors. VG, DG, and BG wrote the first draft of the manuscript with editing and inputs of all authors. All authors designed the experimental setups. All authors contributed to the article and approved the submitted version.

## Funding

This work was funded by the Sonderforschungsbereich 889 of the Deutsche Forschungsgemeinschaft (DFG). VG and DG received a German Federal Scholarship Doctoral Grant awarded by the Deutscher Akademischer Austauschdienst (DAAD). The authors acknowledge the generous support by the Open Access Publication Funds of the Göttingen University. This work was supported financially by a stipend from the German DAAD (VG and DG) and the DFG Collaborative Research Center Cellular Mechanisms of Sensory Processing (SFB-889, A1 to MG).

## Conflict of Interest

The authors declare that the research was conducted in the absence of any commercial or financial relationships that could be construed as a potential conflict of interest.

## Publisher's Note

All claims expressed in this article are solely those of the authors and do not necessarily represent those of their affiliated organizations, or those of the publisher, the editors and the reviewers. Any product that may be evaluated in this article, or claim that may be made by its manufacturer, is not guaranteed or endorsed by the publisher.

## Footnotes

^1^Paul Siefert (2020). ReadCompressedJpegSEQ https://www.mathworks.com/matlabcentral/fileexchange/68341-readcompressedjpegseq, MATLAB Central File Exchange. Retrieved November 9, 2020.

^2^Martin Simonovsky (2020). Ellipse Detection Using 1D Hough Transform (https://www.mathworks.com/matlabcentral/fileexchange/33970-ellipse-detection-using-1d-hough-transform), MATLAB Central File Exchange. Retrieved November 9, 2020.

^3^Joseph Kirk (2021). Dijkstra's Shortest Path Algorithm (https://www.mathworks.com/matlabcentral/fileexchange/12850-dijkstra-s-shortest-path-algorithm), MATLAB Central File Exchange. Retrieved February 22, 2021.
